# Natural history and surgical outcomes of Rathke’s cleft cysts: a Spanish multicenter study

**DOI:** 10.3389/fendo.2024.1413810

**Published:** 2024-06-17

**Authors:** Edelmiro Luis Menéndez-Torre, Alba Gutiérrez-Hurtado, María Dolores Ollero, Ana Irigaray, Patricia Martín, Paola Parra, Inmaculada González-Molero, Marta Araujo-Castro, Cindy Idrobo, María Dolores Moure, Ana Rosa Molina, Betina Biagetti, Pedro Iglesias, Miguel Paja, Rocío Villar-Taibo, Alberto Pena, Almudena Vicente, Fernando Guerrero-Pérez, Fernando Cordido, Anna Aulinas, Manel Mateu, Alfonso Soto

**Affiliations:** ^1^ Department of Endocrinology and Nutrition, Hospital Universitario Central de Asturias, Oviedo, Spain; ^2^ Grupo ENDO, Instituto de Investigación Biomédica del Principado de Asturias (ISPA), Oviedo, Spain; ^3^ Departamento de Medicina, Universidad de Oviedo, Oviedo, Spain; ^4^ Department of Endocrinology and Nutrition, Hospital Universitario de Navarra, Pamplona, Spain; ^5^ Department of Endocrinology and Nutrition, Hospital Universitario La Paz, Madrid, Spain; ^6^ Department of Endocrinology and Nutrition, Hospital Regional Universitario de Málaga, IBIMA Plataforma Bionand, Málaga, Spain; ^7^ Department of Endocrinology and Nutrition, Hospital Universitario Ramón y Cajal, Madrid, Spain; ^8^ Department of Endocrinology and Nutrition, Hospital Universitario de Cruces, Baracaldo, Spain; ^9^ Department of Endocrinology and Nutrition, Hospital Universitario Vall d’Hebrón, Barcelona, Spain; ^10^ Department of Endocrinology and Nutrition, Hospital Universitario Puerta de Hierro, Madrid, Spain; ^11^ Department of Endocrinology and Nutrition, Hospital Universitario de Basurto, Bilbao, Spain; ^12^ Department of Endocrinology and Nutrition, Hospital Universitario Santiago de Compostela, Santiago de Compostela, Spain; ^13^ Department of Endocrinology and Nutrition, Hospital Universitario de Toledo, Toledo, Spain; ^14^ Department of Endocrinology and Nutrition, Hospital Universitari de Bellvitge, Barcelona, Spain; ^15^ Department of Endocrinology and Nutrition, Hospital Universitario de Coruña, Coruña, Spain; ^16^ Department of Endocrinology and Nutrition, Hospital Sant Pau, Barcelona, Spain; ^17^ Department of Endocrinology and Nutrition, Hospital Universitario Virgen del Rocío, Sevilla, Spain

**Keywords:** Rathke’s cleft cyst, pituitary, transsphenoidal surgery, cyst size, visual impairment

## Abstract

Rathke’s cleft cysts (RCC) are a common type of lesion found in the sellar or suprasellar area. They are usually monitored clinically, but in some cases, surgery may be required. However, their natural progression is not yet well understood, and the outcomes of surgery are uncertain. The objective of this study is to evaluate the natural history of Rathke’s cleft cysts in patients who are clinically monitored without treatment, and to determine the outcomes of surgery and the incidence of recurrences over time.

**Design and patients:**

National multicentric study of patients diagnosed of Rathke’s cleft cyst (RCC- Spain) from 2000 onwards and followed in 15 tertiary centers of Spain. A total of 177 patients diagnosed of RCC followed for 67.3 months (6–215) and 88 patients who underwent surgery, (81 patients underwent immediate surgery after diagnosis and 7 later for subsequent growth) followed for 68.8 months (3–235).

**Results:**

The cyst size remained stable or decreased in 73.5% (133) of the patients. Only 44 patients (24.3%) experienced a cyst increase and 9 of them (5.1%) experienced an increase greater than 3 mm. In most of the patients who underwent surgery headaches and visual alterations improved, recurrence was observed in 8 (9.1%) after a median time of 96 months, and no predictors of recurrence were discovered.

**Conclusions:**

Rathke’s cleft cysts without initial compressive symptoms have a low probability of growth, so conservative management is recommended. Patients who undergo transsphenoidal surgery experience rapid clinical improvement, and recurrences are infrequent. However, they can occur after a long period of time, although no predictors of recurrence have been identified.

## Introduction

Rathke’s cleft cysts (RCCs) are benign, non-neoplastic lesions located in the sellar and suprasellar regions. They arise from the remnants of Rathke’s pouch and usually consist of a thin wall enclosing a mucous, gelatinous, or caseous fluid core (1).

RCCs are considered in the differential diagnosis of other cystic lesions in the sellar or suprasellar region, such as craniopharyngiomas, cystic pituitary adenomas, arachnoid cysts, and epidermoid cysts (2). Typically, RCC are diagnosed based on their shape, signal intensity, and enhancement characteristics on magnetic resonance imaging (MRI) (3).

Most of RCCs are small and asymptomatic and often discovered incidentally. The incidence of RCCs at routine autopsy ranges from 5 to 33% (4, 5), however some RCC may grow large enough to cause significant pituitary dysfunction, including Arginine Vasopressin (AVP) deficiency, visual field deficits, headaches, or other neurological symptoms (6–8).

In cases incidentally discovered, little is known about their natural course and factors that could influence such evolution. Therefore, it is unclear how the follow-up of these patients should be carried out, how often, and for how long (8, 9). On the other hand, in those patients who have undergone surgery, there is no clear knowledge of the factors that influence it or who will be affected (10–12).

To evaluate the natural history of Rathke’s cleft cysts in patients who are clinically monitored, and to determine the outcomes of surgery and the incidence of recurrences over time, we retrospectively collected longitudinal clinical data from 258 patients diagnosed with RCCs over the past 20 years in 15 Spanish hospitals.

## Material and methods

A retrospective chart review was conducted on patients diagnosed with RCC between 2003 and 2023 at 15 tertiary hospitals in Spain. The study, known as RCC-SPAIN, was carried out by the Endocrinology Departments. The inclusion criteria were as follows: (1) Radiological diagnosis of Rathke´s cleft cysts (RCC), (2) Follow-up data for patients without surgery for more than 6 months and for patients with surgery for more than 3 months, (3) Available data on clinical, hormonal and radiological cyst characteristics at diagnosis and during follow-up, (4) Pathologic confirmation of RCC in patients who have undergone surgery. A registry was established to gather data on clinical characteristics, such as demographic information, clinical, radiological, and hormonal findings at diagnosis, after surgery, and during follow-up, as well as surgical complications and recurrences. The diagnosis was made based on the radiological characteristics observed on the MRI. Data regarding the cysts size, including the transversal, anteroposterior, and craniocaudal diameters, as well as information on cavernous sinus invasion, and hypointensity or hyperintensity in T1 and T2 sequences were recorded. Pituitary function alterations were evaluated following the Endocrine Society Guidelines (13). The diagnosis was based for GH deficiency on IGF1 levels and for cortisol deficiency on the need for glucocorticoid treatment more than 6 months after surgery.

Small cysts less than 5 mm were excluded from the evaluation, as they are not typically followed in most centers.

Major surgical complications were defined as the development of a permanent neurological deficit (oculomotor or visual impairment), postsurgical meningitis, cerebrospinal fluid (CSF) leakage, and intraoperative or postsurgical bleeding requiring reoperation. Postsurgical AVP deficiency was classified as permanent when there was no recovery after 6 months, and transient if the duration was shorter than 6 months (14).

**Figure 1:**
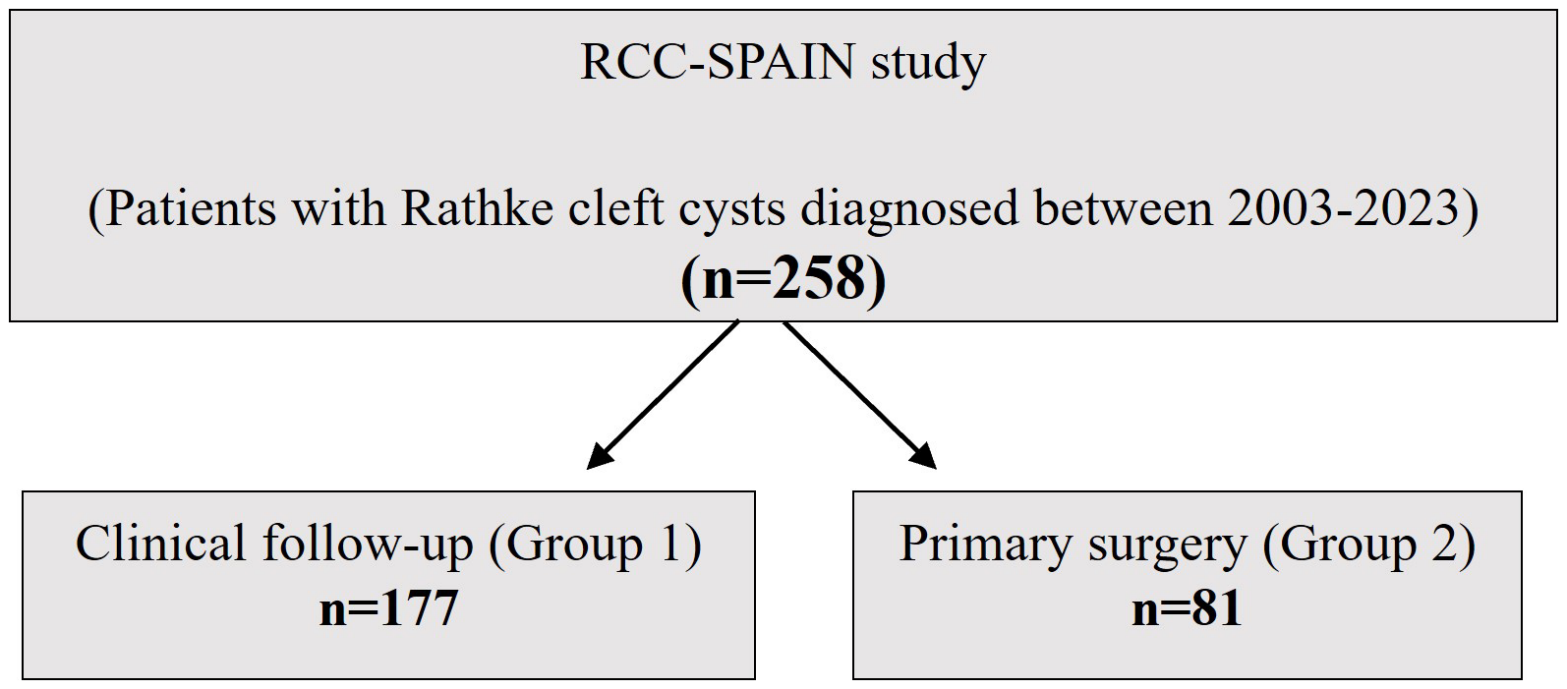
Study population.

Data from 258 patients were analyzed, divided into two cohorts (**Figure 1**). Group 1 consisted of 177 subjects who did not undergo surgery but received clinical, radiological, and hormonal follow-up. Seven of these patients were operated during follow-up. Group 2 comprised 81 subjects who underwent surgical resection of the RCC shortly after diagnosis. The decision to perform surgery was made by the local medical teams on an individual basis, based on the presence of local mass effects such as chiasmal compression, hypopituitarism, and headache.

The study received endorsement from the Spanish Society of Endocrinology and Nutrition (SEEN) and was disseminated to all members of the Neuroendocrinology Task Force of the SEEN, which includes most of the endocrinologists who care for these patients in Spain. The local Ethical Committee of the University Central Hospital of Asturias (HUCA) reviewed and approved the study on February 21^th^, 2023 (N° 2023/092). The study was conducted in accordance with the mandates of the Declaration of Helsinki and good clinical practices. Patient consent was waived due to the retrospective nature of the study.

### Statistical analysis

The statistical analysis was performed with SPSS 27.0. In the descriptive analysis, categorical variables were expressed as percentages and absolute values of variable; quantitative variables were expressed as mean and standard deviation (SD) or as medians and interquartile ranges (IQR) depending on whether the normality assumption was fulfilled. The normality assumption was studied with Shapiro–Wilk test. For the comparison of the differences in continuous parameters between two subgroups we used the Student’s t tests with Bonferroni correction and lineal regression tests. The Chi squared test (with Yates’ correction, when appropriate) was used to compare categorical data. A logistic regression test was performed for multivariate analyses of factors associated with progression of unoperated cysts and with recurrences of cysts after surgery. In all cases, a two-tailed p value < 0.05 was considered as statistically significant.

## Results

### Clinical presentation

There were 258 subjects, 81 men (31.4%) and 177 women (68.6%), who met the inclusion criteria. There were no differences in clinical presentation between men and women but cysts in men had a larger craniocaudal diameter (13.3 ± 8.4 mm) than in women (11.3 ± 6.2 mm; p<0.01).

Non-surgical follow-up was decided in 177 patients (Group 1) and 81 patients underwent transsphenoidal surgery less than 6 months after diagnosis (Group 2).

At diagnosis, the mean age was significantly higher in Group 2 (48.0 ± 16.4 years) compared to Group 1 (42.8 ± 18.1 years) (**Table 1**).

**Table 1:** Demographics and baseline clinical presentation for Group 1 (conservative cohort) and 2 (surgical cohort).

	Total	Group 1	Group 2	p-value
**Number**	258	177 (68.6%)	81 (31.4%)	
**Age (years)**	44.4 ± 17.8	42.8± 18.1	48.0 ± 16.4	0.03
**Men**	81	55 (31.1%)	26 (32.1%)	ns
**Women**	177	122 (68.9%)	55 (67.9%)	ns
**Incidental**	138 (53.5%)	118 (85.5%)	20 (24.7%)	<0.001
**Clinical endocrine dysfunction**	50 (19.4%)	16 (9.0%)	34 (42.0%)	<0.001
**Visual field defects**	44 (17.1%)	3 (1.7%)	41 (50.6%)	<0.001
**Headache**	90 (34.9%)	51 (28.8%)	39 (48.1%)	0.002
**Diplopia**	6 (2.3%)	3 (1.7%)	3 (3.7%)	ns
**Apoplexy**	5 (1.9%)	0 (0%)	5 (6.2%)	–
Diameters in mm (mean (ds)) and volume				
**Transversal**	11.8 (6.1)	9.7 (4.8)	16.5 (6.1)	<0.001
**Craneocaudal**	11.9 (7.0)	9.1 (4.5)	18.1 (7.6)	<0.001
**Anteroposterior**	10.8 (6.4)	8.4 (4.2)	16.1 (7.1)	<0.001
**Volume (mm^3^)**	1.6 (3.2)	0.67 (1.3)	3.6 (4.9)	<0.001
Radiological characteristics				
**Suprasellar extension**	119 (46.1%)	45 (25.4%)	74 (91.4%)	<0.001
**Sinus cavernous extension**	16 (6.2%)	3 (1.7%)	13 (16.3%)	<0.001
**Intracystic hemorrhage**	29 (11.3%)	10 (5.6%)	19 (23.8%)	<0.001
**T1 hypointensity and T2 hyperintensity**	95 (36.8%)	58 (42.0%)	37 (34.1%)	0.211
Pituitary function				
**Any pituitary deficiency**	61 (23.6%)	23 (13.0%)	38 (46.9%)	p<0.001
**TSH deficiency**	35 (13.6%)	12 (6.8%)	23 (28.4%)	p<0.001
**GH deficiency**	25 (9.8%)	8 (4.5%)	17 (21.0%)	p<0.001
**ACTH deficiency**	28 (11.0%)	10 (5.0%)	18 (22.2%)	p<0.001
**LH/FSH deficiency**	43 (16.7%)	16 (9.0%)	27 (33.3%)	p<0.001
**AVP deficiency**	11 (4.5%)	4 (2.2%)	7 (8.6%)	p<0.05
**Hyperprolactinemia**	68 (26.5%)	35 (19.9%)	33 (40.7%)	p<0.001

ACTH, adrenocorticotropic hormone; AVP, Arginine vasopressin FSH, follicle‐stimulating hormone; GH, growth hormone; LH, luteinizing hormone; TSH, Thyrotropin; ns, non significant.

The most common clinical manifestations of RCC at diagnosis were headaches (34.9%), clinical endocrine dysfunction (such as galactorrhea, irregular menses, polyuria, polydipsia, or asthenia; in 19.4%), and visual impairment (17.1%).

As shown in **Table 1**, there was a significant difference in the presenting signs and symptoms as well as pituitary deficiencies, between the two groups. In patients that underwent surgery a higher proportion experienced headaches, visual impairment, and symptoms of endocrine dysfunction. Additionally, an incidental finding of RCC was more common in Group 1, with 85.5% of subjects affected.

The median cyst diameters of the operated patients were 15mm (transversal), 18mm (craneocaudal), and 15mm (anteroposterior). Only one patient had a cyst <10mm which was accompanied by visual field impairments, 42 patients (51,9%) had cysts between 11 and 20mm in diameter while 38 patients (46.9%) had cysts larger than 20mm.

Cyst size and suprasellar extension on MRI correlated significantly with the presence of visual alterations, hypopituitarism, hyperprolactinemia and headaches at diagnosis. There was also a positive correlation between age and size of the cyst. Signal intensity at T1 or T2 did not correlate with any clinical variable.

### Follow-up of not operated patients

Mean follow-up time was 67.3 ± 42.7 months. Out of the 177 patients with data, 132 (74.6%) had stable or decreased largest MRI cyst diameter, while 43 (24.3%) had an increase. Thirty-one patients were followed for less than 2 years, of whom 7 (22.6%) showed cyst growth, 53 patients were followed for 2 to 5 years, of whom 11 grew (20.8%), 72 patients were followed for 5 to 10 years, of whom 21 (29.6%) experienced growth, and 21 patients were followed for more than 10 years, of whom 4 (19%) grew. There were no significant differences in the percentage of growth between the 4 groups.

The increase was only a few millimeters in most cases, and in only 12 patients (6.9%) did it increase by more than 3 mm.

The size change did not differ based on initial size, with 28.3% of RCC less than 10 mm growing and 25.3% of RCC greater than 10 mm growing.

None of the baseline cyst or patients’ characteristics predict the evolution of size after using multiple regression models.

Pituitary function improved in some patients, and in only one case new hormonal changes occur, as shown in **Table 2**. Clinical symptoms, particularly headaches, also improved.

**Table 2:** Patients not operated (177).

	Basal	Final	p-value
**Visual field defects**	3 (1.7%)	1 (0.6%)	ns
**Headache**	51 (28.8%)	24 (13.6%)	<0.001
**Diplopia**	3 (1.7%)	2 (1.1%)	ns
**Any pituitary deficiency**	23 (13.0%)	21 (11.9%)	ns
**TSH deficiency**	12 (6.8%)	13 (7.3%)	ns
**GH deficiency**	8 (4.5%)	12 (6.8%)	0.046
**ACTH deficiency**	10 (5.6%)	9 (5.1%)	ns
**LH/FSH deficiency**	16 (9.0%)	13 (7.3%)	ns
**AVP deficiency**	4 (2.2%)	4 (2.2%)	ns
**Hyperprolactinemia**	35 (19.8%)	25 (14.1%)	ns

ACTH, adrenocorticotropic hormone; AVP, Arginine vasopressin; FSH, follicle‐stimulating hormone; GH, growth hormone; LH, luteinizing hormone; TSH, thyrotropin, ns, non-significant.

During the follow-up period, 7 patients (3.9%) underwent surgery. One patient opted for surgery without any prior changes 3 years after diagnosis, while 3 patients underwent surgery after experienced visual alterations (2 cases) or new pituitary hormone deficiency (1 case). The remaining 3 patients underwent surgery due to significant growth observed in the MRI, which posed a risk of chiasmatic involvement. These surgeries were performed between 27 and 105 months after diagnosis (Median: 67.5 months).

When baseline clinical covariates (Sex, age in years, headaches, visual field alterations, diplopia, pituitary clinical dysfunction, hyperprolactinemia, and presence of suprasellar extension) were included in a logistic regression model, need of surgery was associated with headaches at diagnosis (OR: 11.2; IC 95%: 1.13–110.5) and presence of suprasellar extension of the cyst on MRI (OR: 11.7; IC 95%: 1.37–99.7).

### Surgical results

A total of 88 patients with a diagnosis of RCCs underwent surgery, 58 (65.9%) due to clinical symptoms and 30 (34,1%) for risk of chiasmatic compression. Most surgeries (73 patients) were by endoscopic transsphenoidal approach, 11 by microscopic transsphenoidal approach and only 4 by transfrontal route due to huge size.

Men represented 31.8% of the cases (n=28) and 59 were women. The mean age was 48±19 years for men and 47±16 years for women.

Complete resection was achieved in 61.2% of patients, as defined by detachment of the cyst on post-surgical MRI.

After surgery, headaches improved in most patients (86.4%). Among the 42 patients who had visual field alterations before surgery, 27 (64.3%) normalized their visual fields, 5 (11.9%) showed improvement, 2 (4.8%) showed worsening, and 8 remained unchanged (18.2%).

While hyperprolactinemia persisted in only 11 of 37 patients, the number of patients with hormonal deficiencies increased, mainly due to new cases of arginine vasopressin deficiency (see **Table 3**).

**Table 3:** Evolution of operated patients (n:88).

	Presurgery	Postsurgery	p-value
**Visual field defects**	42 (47.7%)	15 (17.0%)	<0.001
**Headache**	44 (50.0%)	6 (6.8%)	p<0.001
**Diplopia**	3 (3.4%)	2 (2.3%)	ns
**Any pituitary deficiency**	40 (45.5%)	52 (59.1%)	<0.001
**TSH deficiency**	25 (28.4%)	33 (37.5%)	<0.001
**GH deficiency**	19 (21.6%)	26 (29.5%)	<0.001
**ACTH deficiency**	20 (22.7%)	39 (44.3%)	<0.001
**LH/FSH deficiency**	29 (33.0%)	35 (39.8%)	<0.001
**AVP deficiency**	7 (8.0%)	21 (23.9%)	<0.001
**Hyperprolactinemia**	37 (42.0%)	11 (12.5%)	<0.001

ACTH, adrenocorticotropic hormone; AVP.Arginine vasopressin; FSH, follicle‐stimulating hormone; GH, growth hormone; LH, luteinizing hormone; TSH, thyrotropin, ns, non-significant.

Complications were observed in 35.2% of the patients, as shown in **Table 4**. None of the baseline clinical (headaches, previous hypopituitarism) or radiological features on MRI (size, intensity, or suprasellar extension) nor the extent of resection were predictive of the occurrence of new hormone deficiencies or other postoperative complications (meningitis, cerebrospinal fluid fistula, hemorrhage, seal abscess, reintervention, or mortality) after application of logistic regression models.

**Table 4:** Complications of surgery.

Patients with complications	31 (35.2%)
**Cerebrospinal fluid fistula**	10 (11.4%)
**Meningitis**	4 (4.5%)
**Intracranial hemorrhage**	2 (2.3%)
**Transient diabetes insipidus**	21 (23.9%)
**Permanent vasopressin deficiency**	14 (15.9%)
**Increased anterior pituitary dysfunction**	23 (26.1%)
**Sellar abscess**	1 (1.1%)
**Mortality**	1 (1.1%)
**Reintervention**	11 (12.5%)

After a long follow-up (68.7 ± 52.8 months), new cyst growth was observed in 8 patients (9.1%) of the total group. Three patients out of 47 (6.4%) recurred before 5 years of follow up, 3 out of 27 (11,1%) between 5 and 10 years and 2 out of 14 (14,3%) after 10 years. The median time to recurrence after surgery was 96 months and in 4 of the 8 patients it occurred after at least 100 months.

In 4 patients, there was postoperative growth of the remaining tissue, and in another 4, there was a relapse after previous disappearance. The mean growth was 5.9 ± 5.0 mm, with 3 patients experiencing growth of 10 mm or more. Only 1 patient (1.1%) required reoperation due to chiasmatic risk.

A model of bivariate logistic regression for postoperative recurrence or growth was used, including sex, age at diagnosis, initial size and extent, baseline clinical and hormonal data, MRI intensities, total resection, centers surgery load and follow-up time as covariates. None of them were statistically significant for predicting tumor recurrence.

## Discussion

We conducted a multicenter retrospective study observing the evolution of RCC in both patients who did not undergo surgery (group 1) and those who underwent surgery immediately after the diagnosis (group 2).

The baseline data are consistent with previous studies showing a higher prevalence of RCCs in women. The mean age of patients who underwent surgery was 48 years, which was higher than the age of non-operated patients (42 years). This observation has not been reported in other studies (15, 16), probably because the incidental diagnosis of small cysts increased with the increasing number of radiologic examinations performed in recent years. For the same reason, while in Lin et al. (17) and Sala et al. (15), the diagnosis was incidental in about half of the patients, in our case it reached almost 86% in those who did not undergo surgery.

Headache was the most frequently reported symptom at diagnosis, present in 34.9% of all patients and 48.1% of those who underwent surgery, which is consistent with other studies (15, 18). The frequency of hypopituitarism in the series has been highly variable, being this variability likely due to differences in patient composition of the series and of diagnostic protocols. However, a high frequency was observed in our series, with hormonal deficiencies in up to 13% of patients in group 1, and hyperprolactinemia in 26.5%. Similar to other reports (15, 19), the most common hormonal abnormalities were hypogonadism and hyperprolactinemia. Not surprisingly, both pituitary deficiency and hyperprolactinemia were significantly more common in group 2 (patients undergoing surgery) than in group 1.

In terms of size, patients in group 2 had larger diameters, with a significantly higher percentage (91.4%) showing suprasellar extension compared to only 24.9% in group 1. It is worth noting that surgical patients in all series (11, 12, 17, 19–25) had high rates of suprasellar extension (60 to 80%) and visual impairment (up to 56%). In our study the suprasellar extension rate of 91.4% was the highest ever recorded, suggesting that surgery was only recommended for patients with larger cysts who had a higher risk of visual impairment.

The cysts operated on in our study had a larger mean diameter of 18.1 mm, clearly larger than those reported by Sala et al. (15) (12.9 mm) and similar to those reported by Petersson et al. (19) (18.2 mm).

Highly variable data on the natural history of Rathke’s cleft cysts have been published in studies with different follow-up periods and cyst sizes (**Table 5**). After a 9-year follow-up, Aho et al. (21) saw that in 31% of these patients the cyst progressed over time, causing visual impairment and/or pituitary dysfunction and were therefore operated. Culver et al. (27) found that the majority of radiologically diagnosed RCCs remained unchanged (57%) or decreased in size (15%) after a median of 24 months, suggesting that in the absence of pressure symptoms, it is reasonable to manage these patients conservatively. More recent studies have shown growth of only 6.5% at 57 months (15) or 10% at 36 months (8) requiring re-operation 2.2% in the latter case. Peterson et al. (19) found that patients with cysts smaller than 10 mm rarely progressed, as did those who did not undergo surgery in the first year, even if they were larger.

**Table 5:** Natural history of RCCs in different series.

	Number of patients	Median age (years)	Median size (mm)	Growth	Reduction	Follow-up time (Median and range in months)	Operated
Sala et al. (15)	62	46	7.9	6.4%	3.2%	41 (6–143)	0%
Culver et al. (26)	75	41.3	16	28%	15%	24 (1–126)	25%
Aho et al. (21)	61	—		31.1%	—	108 (60–108)	31%
Petersson et al. (19) (>10 *mm*)	174	46.1	13.5	15%	48%	60	—
Menéndez-Torre et al. (8)	229	43	10	10%	31.9%	36.6 (1–210)	2.2%
Menéndez et al	177	42.8	9.7	24.3%	25,4%	65 (6–215)	3%

In our case, after a mean follow-up of more than 65 months, a quarter increased in size, but most of them were clinically meaningless and clinical symptoms and hormonal alterations either remained stable or decreased in the vast majority of patients as in other series (11, 17, 19). Many attempts have been made to determine the characteristics of those patients with cysts who are at higher risk of growth. Peterson et al. (19) suggested that those smaller than 10 mm did not grow, Kinoshita (8) identified older age as the only factor influencing progression and Kim et al. (26) identified a subgroup of cysts with T1 hypointensity and T2 hyperintensity on RMI with higher risk of growth. In our series the multivariate analysis including these factors did not reveal any clinical or cyst characteristics that influenced cyst growth over time. Only 6 cases (3%) required surgery and it was between 27 and 105 months after diagnosis with a median time of 65.7 months. Predictors of the need for surgery in these patients were the presence of headache and suprasellar extension at diagnosis.

The surgical treatment results in our series are excellent, with many patients experiencing improvement in visual impairment, and in headache. These findings confirm the results of recent studies (11, 19, 28), with patients operated on through transsphenoidal route as in our case. After surgery, hyperprolactinemia improves in a high percentage of patients due to decompression of the pituitary stalk. However, pituitary hormone deficiency does not always improve and may even worsen in some patients, as reported by other authors and a more conservative surgical approach would probably be advisable (15, 19, 29, 30).

Methodological differences between studies may account for the wide range in recurrence rates observed, from 0 (20) to nearly 30% (17). A meta-analysis of 1151 cases found a recurrence rate of 12.5% of recurrences (31)after 38 months of follow-up on average, although the range of follow-up was 16 – 79 months. In our study the recurrence rate was low (9.1%) after a longer follow-up (68.7 months) and a follow-up between 3 and 238 months. Recurrences were observed in many cases after long follow-up, as shown in a systematic review that demonstrated an increasing incidence over time and a significant rise after a follow up of more than 72 months (32).

Numerous predictors of recurrence have been described, including enhancement on MRI, extent of cyst resection, presence of residual cyst, inflammatory change, squamous metaplasia, use of alcohol irrigation, preoperative size, fat grafting, and transitional histology, but Kim et al. (29) could not determine any statistical parameters associated with increased risk of recurrence. Residual cyst on postoperative MRI is associated with an increased risk of recurrence (11) and Billeci et al. (33) suggested that recurrence or relapse is probably more often due to incomplete surgical removal. We did not detect any characteristics associated with the risk of recurrence after assessing age, sex, initial size and extent, centers load of surgery, baseline clinical and hormonal data, MRI intensities and residual cyst persistence.

Limitations of this study include its retrospective design and multicentric nature. In addition, the diagnosis of a RCC was based on the characteristic appearance on MRI, so we cannot exclude the possibility that some of the patients in the nonsurgical group had cysts other than RCC.

Our study concludes that Rathke’s cleft cysts without initial compressive symptoms have a low probability of growth. Therefore, these cases should be managed conservatively with periodic MRI. Patients who undergo transsphenoidal surgery experience rapid clinical improvement with still a high complication rate. Although recurrences are rare, they may occur after a long period of time. No predictors have been identified, and further studies are needed. Meanwhile long-term clinical and radiological follow-up after surgery is necessary.

## Data availability statement

The original contributions presented in the study are included in the article/supplementary material. Further inquiries can be directed to the corresponding author.

## Ethics statement

The studies involving humans were approved by Comité de Ética de la Investigación del Principado de Asturias (Hospital Universitario Central de Asturias N-1, S3.19 Avda. de Roma, s/n 33011 - Oviedo). The studies were conducted in accordance with the local legislation and institutional requirements. Written informed consent for participation was not required from the participants or the participants’ legal guardians/next of kin in accordance with the national legislation and institutional requirements.

## Author contributions

ET: Conceptualization, Data curation, Investigation, Methodology, Software, Supervision, Visualization, Writing – original draft, Writing – review & editing, Formal analysis, Funding acquisition, Project administration, Resources, Validation. AH: Conceptualization, Writing – original draft, Writing – review & editing. MO: Supervision, Writing – original draft, Writing – review & editing. AI: Supervision, Writing – original draft, Writing – review & editing. PM: Supervision, Writing – original draft, Writing – review & editing. PP: Supervision, Writing – original draft, Writing – review & editing. IM: Supervision, Writing – original draft, Writing – review & editing. MA: Supervision, Writing – original draft, Writing – review & editing. CI: Supervision, Writing – original draft, Writing – review & editing. MM: Supervision, Writing – original draft, Writing – review & editing. AM: Supervision, Writing – original draft, Writing – review & editing. BB: Supervision, Validation, Writing – original draft, Writing – review & editing. PI: Supervision, Writing – original draft, Writing – review & editing. MP: Supervision, Writing – original draft, Writing – review & editing. RV: Supervision, Writing – original draft, Writing – review & editing. AP: Supervision, Writing – original draft, Writing – review & editing. AV: Supervision, Writing – original draft, Writing – review & editing. FG: Supervision, Writing – original draft, Writing – review & editing. FC: Software, Supervision, Writing – original draft, Writing – review & editing. AA: Supervision, Writing – original draft, Writing – review & editing. MM: Supervision, Writing – original draft, Writing – review & editing. AS: Supervision, Writing – original draft, Writing – review & editing.
